# Mice Recognise Mice in Neighbouring Rearing Cages and Change Their Social Behaviour

**DOI:** 10.1155/2024/9215607

**Published:** 2024-01-16

**Authors:** Hiroshi Ueno, Yu Takahashi, Sachiko Mori, Shinji Murakami, Kenta Wani, Yosuke Matsumoto, Motoi Okamoto, Takeshi Ishihara

**Affiliations:** ^1^Department of Medical Technology, Kawasaki University of Medical Welfare, Okayama 701-0193, Japan; ^2^Department of Psychiatry, Kawasaki Medical School, Kurashiki 701-0192, Japan; ^3^Department of Neuropsychiatry, Graduate School of Medicine, Dentistry and Pharmaceutical Sciences, Okayama University, Okayama 700-8558, Japan; ^4^Department of Medical Technology, Graduate School of Health Sciences, Okayama University, Okayama 700-8558, Japan

## Abstract

Mice are social animals that change their behaviour primarily in response to visual, olfactory, and auditory information from conspecifics. Rearing conditions such as cage size and colour are important factors influencing mouse behaviour. In recent years, transparent plastic cages have become standard breeding cages. The advantage of using a transparent cage is that the experimenter can observe the mouse from outside the cage without touching the cage. However, mice may recognise the environment outside the cage and change their behaviour. We speculated that mice housed in transparent cages might recognise mice in neighbouring cages. We used only male mice in this experiment. C57BL/6 mice were kept in transparent rearing cages with open lids, and the cage positions were maintained for 3 weeks. Subsequently, we examined how mice behaved toward cagemate mice, mice from neighbouring cages, and mice from distant cages. We compared the level of interest in mice using a social preference test. Similar to previous reports, subject mice showed a high degree of interest in unfamiliar mice from distant cages. By contrast, subject mice reacted to mice from neighbouring cages as familiar mice, similar to cagemate mice. This suggests that mice housed in transparent cages with open lids perceive the external environment and identify mice in neighbouring cages. Researchers should pay attention to the environment outside the mouse cage, especially for the social preference test.

## 1. Introduction

Mice have been the most widely used laboratory animals for the study of disease, behaviour, and pharmacology over the past century [1]. Laboratory mice currently play a central role as animal models of human behavioural disorders [2]. Many laboratories worldwide use genetically defined mouse strains and mutant mice to answer complex questions regarding behaviour, which represents the final output of the nervous system in an organism as a result of the interaction between genotype and environment. C57BL/6 mice are a popular mouse strain in behavioural and genetic studies and are used as a standard strain for comparison with other mice [3, 4]. The interest in the reproducibility of behavioural phenotypes in mice is growing [5]. To successfully transfer the results obtained using mice to human experiments, it is necessary to clarify as much as possible the proper treatment, handling, and housing of laboratory mice.

The reproducibility of experimental work in biomedical research has been a hot topic fuelling intense debate over the past decade [6, 7]. The estimated prevalence of irreproducible findings is in the range of 50–90% [8]. In general, phenotypic differences between inbred strains have been suggested to stabilise within laboratories, whereas behaviours related to emotional, cognitive, and social processes are unstable between laboratories [9, 10]. Phenotypes among inbred strains can be influenced by laboratory-specific husbandry and testing parameters [11]. Previous studies have demonstrated how the experimenter and laboratory environment can explain the variability between replicates within and between laboratories [12]. It has been convincingly shown that experimenters [13, 14] and processing methods [15] can be key factors contributing to data variability in behavioural neuroscience. Mouse cage and animal room environments have also been shown to contribute to variation in behavioural neuroscience data [10, 16].

With the recent increase in interest in animal ethics, the focus on improving the housing environment and welfare of laboratory animals is growing within the international laboratory animal research community [17]. Research related to the behavioural analysis of laboratory animals has also reported that rearing conditions, such as group and cage sizes, are important factors influencing animal behaviour [18–20]. Laboratory mouse housing conditions are primarily determined by economics (minimum use of space, equipment, and labour), ergonomics (ease of handling, animal visibility), hygiene (easiness of disinfection), and standardisation [21, 22]. Mice are usually housed in transparent “shoe-box” cages containing bedding, food, and water. Numerous studies have investigated the effects of cage size and housing density on various physiological and behavioural parameters in mice. However, there is little consensus on whether proper housing arrangements generally provide particular advantages or disadvantages with respect to animal use and welfare [23]. Another example of an important housing variable is cage colour. This can affect the anxiety levels experienced by the mice and increase the number of times they attempt to escape from their cages or handlers. Mice tend to prefer opaque cages and seem to breed better in these cages [24, 25], but clear plastic cages have become standard in recent years. Transparent cages have the advantage that the experimenter can observe the animals without having to remove them from the cage or rack. On the other hand, mice in transparent cages may be observing the environment outside the cage as well. We speculated that the animal's perception of the environment outside the cage influences its behaviour. Thus, this study is aimed at determining whether mice in neighbouring cages recognise each other.

Although mice are not primarily guided by vision, many behavioural tests of cognitive function in mice use primarily visual stimuli as cues (open field, elevated plus maze, light-dark box, Morris water maze, radial arm maze, Barnes maze, etc.), and the performance in these tasks depends on the visual ability. The murine visual system performs a variety of functions, from predator detection to finding refuge and selecting food and mates, and needs to do so in diverse environments [26]. The field examining the mouse visual system has garnered a great deal of attention in recent years due to the wealth of tools available for brain circuit dissection in mice, and many groups have adopted mice as models for research on visual perception and vision-based decision-making [27, 28]. Mice can perform hundreds of trials on an operant visual task similar to that used in primates and generate comparable psychophysical data [29] and can be trained to use visual stimuli to navigate virtual reality environments [30]. They use visual and olfactory information to show interest in the abnormal behaviour of others [31–33] and visually recognise cagemates that exhibit abnormal behaviours [34]. These reports suggest that mice may be able to recognise mice in neighbouring cages.

This study is aimed at examining whether mice housed in transparent cages recognise mice in neighbouring cages. After holding the cage positions for 3 weeks, we evaluated the behaviour of subject mice against (1) mice in the same cage, (2) mice in neighbouring cages, and (3) mice in distant cages. This research will contribute to improving the reproducibility of preclinical behavioural science in mice and to discovering new cognitive abilities in mice.

## 2. Results

### 2.1. Degree of Motivation toward Stranger, Neighbour, and Cagemate Mice

We investigated whether the motivation for social behaviour toward a neighbour is equivalent to an interest toward a stranger mouse.

**Figure 1:**
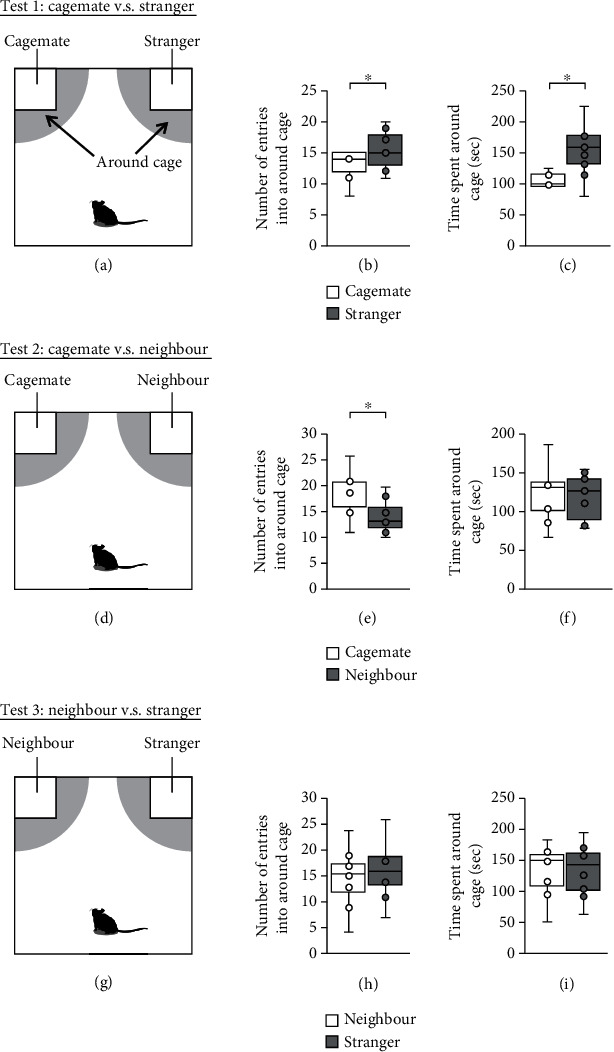
Preference tests for cagemate, neighbour, and stranger mice in the social interaction test apparatus. (a, d, g) Schematic diagram of the apparatus of this experiment. Two transparent cages are placed at both ends of a square apparatus. A radius of 18 cm around the transparent cage was set around the cage (around cage). For each mouse, three tests were conducted according to the figure. Cagemates, neighbours, and strangers mice were placed in transparent cages. Preference tests for cagemate and stranger mice: number of entries into around cage (b) and time spent around the cage (c). Preference tests for cagemate and neighbour mice: number of entries into around cage (e) and time spent around the cage (f). Preference tests for neighbour and stranger mice: number of entries into around cage (h) and time spent around the cage (i). All data are presented as box plots. The *p* values were calculated using paired *t*-tests. *n* = 10 animals per trial. (b) *t* = −2.34, (c) *t* = −3.655, (e) *t* = −3.175, (f) *t* = −0.735, (h) *t* = −0.697, and (i) *t* = −0.021.  ^∗^*p* < 0.05,  ^+^*p* < 0.05.

First, both stranger and cagemate mice were placed in transparent cages in the corners of the apparatus (Figure 1(a)). The number of entries of subject mice in the area around the cage containing the stranger mouse was greater than that around the opposite-positioned cage with the cagemate mouse (Figure 1(b), *t* = −2.340, *p* = 0.047^∗^, paired *t*-test). Moreover, subject mice showed a preference for spending time around the transparent cage with the stranger mouse (Figure 1(c), *t* = −3.654, *p* = 0.006^∗^, paired *t*-test). Next, neighbour and cagemate mice were placed in the two transparent cages (Figure 1(d)). The number of entries of subject mice in the area around the cage containing the cagemate mouse was greater than that around the opposite-positioned cage with the neighbour mouse (Figure 1(e), *t* = −3.175, *p* = 0.013^∗^, paired *t*-test). No significant differences were found between the time spent around the cage with the cagemate mouse and that around the opposite-positioned cage with the neighbour mouse (Figure 1(f), *t* = −0.735, *p* = 0.483, paired *t*-test). Finally, both neighbour and stranger mice were placed in the two transparent cages (Figure 1(g)). No significant differences were found between the number of entries into the area around the cage with the neighbour mouse and that around the opposite-positioned cage with the stranger mouse (Figure 1(h), *t* = −0.697, *p* = 0.51, paired *t*-test). Likewise, no significant differences were found between the time spent around the cage with the neighbour mouse and the time spent around the opposite-positioned cage with the stranger mouse (Figure 1(i), *t* = −0.020, *p* = 0.984, paired *t*-test).

**Figure 2:**
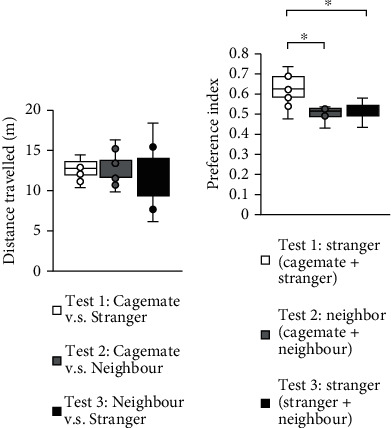
Total distance travelled and preference index for preference tests for cagemate, neighbour, and stranger mice in the social interaction test apparatus. (a) Total distance travelled for preference tests. (b) Preference index for preference tests. Preference index defined as (time spent around cage (a))/(time spent around cage (a) + time spent around cage (b)). All data are presented as box plots. One-way ANOVA, followed by Tukey's test, was used for statistical analysis: (a) *F*_2,24_ = 0.492; (b) *F*_2,24_ = 7.048. *n* = 10 animals per trial.  ^∗^*p* < 0.05,  ^+^*p* < 0.05.

No significant difference was detected between the three conditions in terms of distance travelled (Figure 2(a); test 1 vs. test 2: *p* = 0.812; test 1 vs. test 3: *p* = 0.347; test 2 vs. test 3: *p* = 0.475, *F*_2,23_ = 0.492, ANOVA). However, we found significant differences in the preference indices between the three conditions (Figure 2(b)). The preference index of test 1 (stranger/(cagemate + stranger)) was significantly higher than that of test 2 (neighbour/(cagemate + neighbour)) (Figure 2(b), *p* = 0.002^∗^, *F*_2,23_ = 7.048, ANOVA). Moreover, the preference index of test 1 (stranger/(cagemate + stranger)) was significantly higher than that of test 3 (stranger/(neighbour + stranger)) (Figure 2(b), *p* = 0.007^∗^, ANOVA). No significant difference was found between the preference index in test 2 and the preference index in test 3 (Figure 2(b), *p* = 0.674, ANOVA).

### 2.2. Social Behaviour toward Stranger, Neighbour, and Cagemate Mice

**Figure 3:**
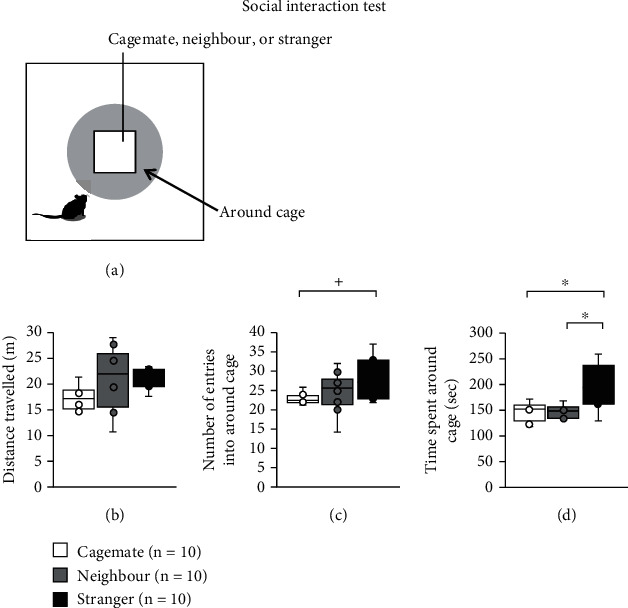
Social interaction test. (a) Schematic diagram of the social interaction test showing the position of the transparent cage and the interaction zone (around cage). Total distance travelled (b), number of entries around cage (c), and time spent around cage (d) under cagemate, neighbour, and stranger mouse conditions. All data are presented as box plots. One-way ANOVA, followed by Tukey's test, was used for statistical analysis: (b) *F*_2,24_ = 1.044; (c) *F*_2,24_ = 1.951; (d) *F*_2,24_ = 3.741. The *p* values were calculated using one-way ANOVA. *n* = 10 animals per trial.  ^∗^*p* < 0.05,  ^+^*p* < 0.05.

The subject mice were presented with one cagemate, one neighbour, or one stranger mouse to determine their social behaviour (Figure 3(a)). In test 1, a cagemate mouse was placed in the central transparent cage. In test 2, a neighbour mouse was placed in the transparent cage. In test 3, a stranger mouse was placed in the transparent cage. The subject mouse was placed in the corner of the apparatus and allowed to freely explore the entire box for 10 min. No significant difference was detected between the three conditions in the distance travelled (Figure 3(b); cagemate vs. neighbour: *p* = 0.199; cagemate vs. stranger: *p* = 0.268; neighbour vs. stranger: *p* = 0.959, *F*_2,23_ = 1.044, ANOVA). The number of entries of the subject mice in the area around the cage containing the stranger mouse tended to be higher than that around the cage containing the cagemate mouse (Figure 3(c), *F*_2,23_ = 1.951, *p* = 0.066^+^, ANOVA). No significant differences were found in the number of entries in the area around the cage with the cagemate mouse and that around the cage with the neighbour mouse (Figure 3(c), *p* = 0.382). Similarly, no significant difference was found between the number of entries in the area around the cage with the neighbour mouse and those around the cage with the stranger mouse (Figure 3(c) , *p* = 0.231). The time spent around the cage with the stranger was significantly higher than the time spent around the cage with the cagemate (Figure 3(d), *p* = 0.030^∗^, *F*_2,23_ = 3.741, ANOVA). The time spent around the cage with the stranger was significantly higher than the time spent around the cage with the neighbour (Figure 3(d) , *p* = 0.024^∗^, ANOVA). No significant difference was found between the time spent around the cage with the cagemate mouse and that around the cage with the neighbour mouse (Figure 3(d) , *p* = 0.963, ANOVA).

## 3. Discussion

This study investigated whether mice in transparent cages can recognise mice in neighbouring cages. We hypothesised that mice housed in transparent cages perceive the environment outside the cage and change their behaviour. In this experiment, mice housed in transparent cages showed different social behaviours toward those in neighbouring cages and those in stranger cages.

In the social preference test, the subject mice were highly interested in stranger mice. Interest in neighbour mice was similar to that in cagemates. Mice and rats, the primary experimental mammalian models used in biomedical research, are social species that exhibit social cognition [35–37]. Mice are highly social animals, and in the wild, they usually live in family groups consisting of a dominant male, several females and their offspring, and subordinate males [38]. Mice spend more time exploring social stimuli than inanimate objects and show a preference for new conspecifics over familiar ones [39]. Specifically, during social interactions, these animals exhibit higher investigative behaviour toward unfamiliar or novel conspecific individuals (hereafter referred to as social stimuli) compared to familiar individuals [40]. Thus, in the social discrimination tests, subject mice spent less time exploring familiar stimuli than novel conspecific individuals, reflecting their perceptions of familiar stimuli. This type of social cognition is frequently used in social neuroscience to assess typical social behaviour [41, 42]. In the sociability test of the current study, subject mice also showed increased interest in unfamiliar mice compared to their cagemates and neighbours. Furthermore, the interest of subject mice in neighbour and cagemate mice was comparable. The results of this study suggest that mice housed in transparent cages recognise mice in neighbouring cages as familiar.

Mice use visual cues for important behaviours, such as hunting, avoidance, and navigation, but are much less dependent on the visual system [43, 44]. Mice may use fine visual perception to discriminate between complex nonsocial visual stimuli. Although operant experiments have traditionally favoured rats, touchscreen technology has provided researchers with a new tool for examining visual cognition in mice [45, 46]. Mice can perceive virtual reality spaces [47, 48] and can visually distinguish photographs [49]. The Morris water maze test is a widely used model to study learning and memory in mice. This test specifically assesses spatial learning and memory [50, 51] and relies on distal cues to identify submerged escape platforms from starting positions around a swimming pool. In other words, it is assumed that the mouse can visually grasp the surrounding environment while floating on water. Moreover, mice visually grasp the actions of other individuals and perform empathy- and mimicry-like behaviours [52–56]. It is possible that olfactory information from mice in neighbouring cages might have affected the mice used in this study [57]. However, in this study, the top of the cage was covered with a nonwoven filter top. Therefore, it is assumed that there is little olfactory information from neighbouring cages. The subject mice mainly recognised the mice in neighbouring cages using visual and olfactory information and responded to them as familiar individuals.

Behavioural experiments in mice have long been an important test and are widely practised worldwide [46, 58]. It has been reported that most behavioural traits are sensitive to genetic, environmental, and experimental factors, such as genetic background, laboratory conditions, and previous testing experience [9, 12]. In a series of behavioural experiments, it is necessary for the experimenter to conduct the experiments appropriately. However, in many publications, it is not possible to deduce from Materials and Methods the environment and method of the behavioural experiments, and different laboratories often have diverging experimental results. Before being used in experimental procedures, laboratory mice spent most of their lives in their home cages. Environmental conditions within animal facilities can have a significant impact on the health of rodents used in behavioural experiments, especially on tests that measure spontaneous behaviour [59]. The results of the current study also suggest this as a possibility for why laboratories obtain different experimental results. Mice used as stimuli in social preference testing must be age-, sex-, and strain-matched but foreign to the test mouse. Cagemates of test mice should not be used as stimulator mice [60]. Our research results suggest that neighbour mice should also not be presented as the stimulus.

The use of only male mice is a limitation of our study. The main purpose of this study was to investigate how mice housed in transparent cages perceive mice in neighbouring cages as their external environment. Further studies are needed to determine whether female mice perceive neighbouring cage situations as well as male C57BL/6N mice. In this study, we used C57BL/6 mice. C57BL/6 mice are widely used as an inbred strain for knockout and transgenic models [61]. C57BL/6 and DBA/2J mice are the oldest and most commonly used inbred strains in behavioural genetics. Many behavioural domains are thought to exhibit a moderate phenotype [41], which allows the detection of behavioural changes at baseline and in response to various manipulations [62, 63]. In particular, the C57BL/6 strain is thought to have superior spatial memory [64, 65]. However, it is speculated that the perception of the environment outside the cage differs depending on the mouse strain used in the experiment [66]. AKR and DBA mouse strains have been reported to exhibit good visual acuity [67, 68]. Further studies are needed to clarify mouse strain and sex differences in the perception of the environment outside the cage. We also need to consider experiments in which animals are kept in opaque cages.

## 4. Conclusions

We found that mice housed in transparent cages recognise mice in neighbouring cages and show behaviour toward them similar to that toward their cagemates. This study reports not only visual stimuli but also other sensual stimuli that may have contributed to the observed recognition of conspecifics. The study results further emphasise the necessity of adjusting the rearing environment of mice, particularly for the social preference test.

## 5. Materials and Methods

### 5.1. Ethics Statements

All animal experiments were performed in accordance with the ARRIVE guidelines (https://www.nc3rs.org.uk/arrive-guidelines) and the U.S. National Institutes of Health (NIH) Guide for the Care and Use of Laboratory Animals (NIH Publication No. 80-23, revised in 1996). This study was approved by the Committee for Animal Experiments at the Kawasaki Medical School Advanced Research Centre. All efforts were made to minimise the number of animals used and their suffering. The use of animals was minimised via an experimental design that permitted statistically significant changes to be demonstrated with the smallest number of animals per group and the smallest number of groups, consistent with scientific rigour. This study used a factorial design based on group size. A priori sample size was determined using Mead's rule [69]. Based on Mead's equation and the law of diminishing returns [69], this sample size was large enough for sufficient error degrees of freedom.

### 5.2. Animals

We obtained 8-week-old 125 C57BL/6NJcl inbred mice from CLEA (Tokyo, Japan) in total. They were housed in transparent plastic cages (220 mm × 340 mm × 150 mm, five animals per cage) with wire tops. A nonwoven filter cap was attached on top of the wire top. The cages included the provision of nesting material with food (MF-R; ORIENTAL YEAST, Tokyo, Japan) and water ad libitum, under 12 h light/dark conditions (lights on at 8:00, lights off at 20:00), a temperature maintained between 23 and 26°C, the illuminance of 140 lx during the light period, and at a relative humidity of 40–50%. Considering that behavioural variability is partially sex-dependent and that comparing the behaviour of males vs. females was not the purpose of this experiment, only male mice were included in this study. To prevent aggression or fighting that may occur, we excluded mice that exhibited such behaviours. In this experiment, no mice showed aggressive or fighting behaviour. Male mice were also selected to further elucidate the reproducibility of previous behavioural experiments using male mice [70].

### 5.3. Rearing Conditions

**Figure 4:**
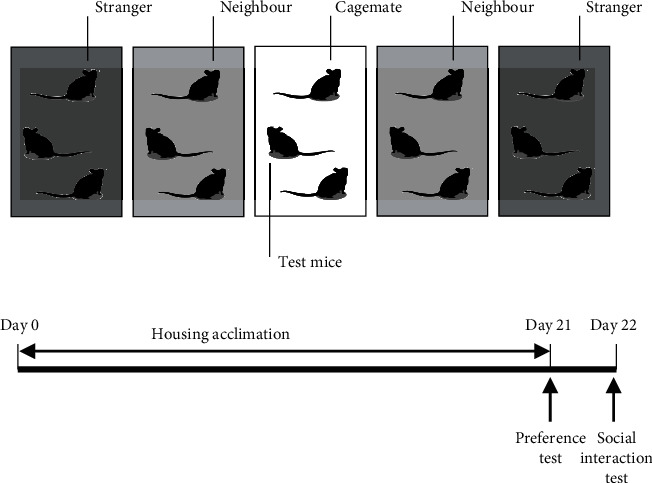
Positional relationship of breeding cages in breeding environment and experimental design. (a) Mice in the same cage as the test mice are the cagemates, next to the cage are neighbours, and mice two next to the cage are strangers. (b) Experimental time schedules. For 3 weeks, the housing cages containing the mice remained in the same location. Afterwards, we performed behavioural tests.

The mice were randomly (https://www.randomizer.org) divided into three groups: stranger, neighbour, and cagemate groups (Figure 4(a)). Subject mice (*n* = 10 per trial) were randomly selected from the cagemate groups. We selected 2 mice out of 5 cages as subject mice. Over the course of three weeks, the cages housing the mice used in this experiment were kept in the same position and height (Figure 4(b)). These rearing cages were not moved except for cleaning.

### 5.4. Behavioural Tests

All behavioural tests were conducted in behavioural testing rooms between 09:00 and 16:00, during the light phase of the light/dark cycle. Tests were separated by 24 h. The mice were tested in random order. After the tests, the equipment was cleaned with 70% ethanol and super hypochlorous water to prevent artefacts caused by lingering olfactory cues. Behavioural tests were performed on naïve mice according to the test protocols described below.

### 5.5. Preference Tests for Cagemate, Neighbour, and Stranger Mice

In this test, we used randomly selected naïve mice that were not used in other tests. The square-shaped apparatus had a size of 42 cm × 42 cm × 40 cm. Two transparent cages (7.5 cm × 7.5 cm × 10 cm, with several holes of 1 cm diameter each) were placed at two ends of this apparatus (Figures 1(a), 1(d), and 1(g)). The design allowed nose contact between the bars but prevented the mice from fighting. Each mouse was placed in their box and allowed to freely explore for habituation for 10 min. In test 1, a cagemate mouse was placed in one transparent cage, and a stranger mouse was placed in the other transparent cage (Figure 1(a)). In test 2, a cagemate mouse was placed in one transparent cage, and a neighbour mouse was placed in the other transparent cage (Figure 1(d)). We randomly selected neighbouring mice from the cage to the right or left of the subject mouse. In test 3, a neighbour mouse was placed in one transparent cage, and a stranger mouse was placed in the other transparent cage (Figure 1(g)). We randomly selected stranger mice from the cage to the right or left of the subject mouse. The subject mouse was placed at the centre of the apparatus and allowed to freely explore the entire box for 10 min. The number of entries into the vicinity of each cage and the amount of time spent near each cage during the 10-minute sessions were measured. We also analysed the total distance travelled in each trial. Preference index for preference tests. Preference index is defined as follows: (time spent around cage (a))/(time spent around cage (a) + time spent around cage (b)). The data were recorded on video and analysed using the ANY-maze software. The apparatus was cleaned after each phase of this test.

### 5.6. Social Interaction Test

Social interaction behaviour was investigated using the apparatus described for the preference test but containing only one transparent cage (7.5 cm × 7.5 cm × 10 cm, with several holes of 1 cm diameter; Figure 3(a)). Each mouse was placed in a box and allowed to freely explore for habituation for 10 min. In test 1, a cagemate mouse was placed in the central transparent cage. In test 2, a neighbour mouse was placed in the transparent cage. In test 3, a stranger mouse was placed in the transparent cage. The subject mouse was placed in the corner of the apparatus and allowed to freely explore the entire box for 10 min. We recorded the amount of time each mouse spent interacting with the transparent cage. We also analysed the total distance travelled in each trial. The apparatus was cleaned after each phase of this test. In this test, we used naïve mice that were not used in other tests. The data were recorded on video and analysed using the ANY-maze software.

### 5.7. Statistical Analyses

Statistical analysis was conducted using the SPSS software (IBM Corp., Tokyo, Japan). Normal distribution was determined with the Shapiro–Wilk normality test for all samples before any group analysis. For normally distributed paired samples, we used a paired *t*-test. For not normally distributed paired samples, we used a Mann–Whitney *U* test. We used one-way analysis of variance (ANOVA) followed by Tukey's test to compare three experimental groups in which unpaired samples were normally distributed. We used the Kruskal-Wallis to compare three experimental groups in which unpaired samples were not normally distributed. Data are presented as box plots. Statistical significance was defined as ^∗^*p* < 0.05 and ^+^*p* < 0.1.

## Data Availability

The datasets generated and analysed during the current study are not publicly available. However, these data may be made available by the corresponding author upon reasonable request.
